# Cubes or Pellets in Mental-Rotation Tests: Effects on Gender Differences and on the Performance in a Subsequent Math Test

**DOI:** 10.3390/bs10010012

**Published:** 2019-12-23

**Authors:** Martina Rahe, Claudia Quaiser-Pohl

**Affiliations:** Institute of Psychology, University of Koblenz-Landau, 56070 Koblenz, Germany; quaiser@uni-koblenz.de

**Keywords:** mental rotation, gender differences, stimulus material, confidence, math test, gender stereotypes

## Abstract

In mental rotation, males consistently outperform females in performance and confidence. Both can affect math anxiety. In the present study, 107 undergraduate students (85 female) solved a mental-rotation test either with cube (C-MRT) or pellet (P-MRT) figures as stimulus material, then reported their confidence in their ability in the test, and solved a math test. Males performed better than females in both test versions: In the C-MRT, with a large effect, and in the P-MRT, with a small effect, and reported higher scores in their confidence. In math test performance, males scored higher than females when they solved the math test after the C-MRT but not after the P-MRT. The interactions of gender and stimulus material were not significant. Correlations between confidence and math test performance were large for males and not significant for females. Stereotype threat and lift effects are discussed as possible reasons for the varying effects of the stimulus material on the MRT performance of male and female participants.

## 1. Introduction

Spatial ability is defined as “representing, transforming, generating, and recalling symbolic, nonlinguistic information” ([1], p. 1482) and is required in many school and working settings, e.g., geometry, architecture, aviation, chemistry, or sports science. Spatial abilities are often divided into the categories, spatial perception, mental rotation, and spatial visualization [1]. Mental rotation is defined as the ability to rotate objects quickly and accurately in the mind [2].

In mental-rotation tests, gender differences in favor of males consistently appear [2], with large effect sizes, especially for paper-and-pencil tests (mental rotation test, MRT, according to [3]). Explanations for these gender differences could be biological [4], psychosocial [5], or strategic [6] as well as related to task characteristics [7,8,9,10]. The complexity or familiarity of the stimulus material [7,8] or difficulty and rotational axis [9,10] had effects on mental-rotation performance in general or the gender differences in these performances. 

**Figure 1:**
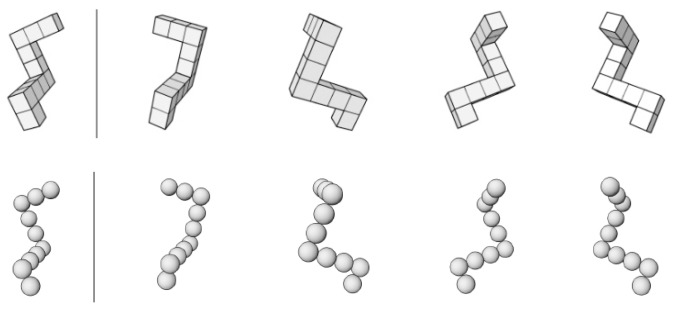
Example items (C-MRT in the upper row and P-MRT in the lower row).

Another important aspect is the gender-stereotyped nature of the rotational objects. Previous studies with gender-stereotyped objects as stimulus material, e.g., cars or dolls, found a significant interaction of participants’ gender and rotational objects for fourth graders [11] and elderly participants [8]. Using cube (C-MRT) and pellet (P-MRT) figures (Figure 1) instead of actual objects in fourth graders, gender differences in mental-rotation performance in favor of boys were found for the C-MRT but were absent for the P-MRT [12]. Similar results were found in a study using the same material [13]. In both studies, cube figures were perceived as male stereotyped and pellet figures as female stereotyped [12,13], probably because cube figures resemble construction toys while pellet figures resemble a necklace.

So, reasons for better performances when using own gender-stereotyped objects as stimulus material could be implicit stereotype threat [14] and stereotype lift [15] effects. Stereotype threat is defined as being at risk of confirming to an existing negative stereotype about one’s own group [14] while stereotype lift is the belief to belong to a group that has higher abilities in a certain area [15]. Both effects can influence performance; manipulation with a stereotype threat can impair the performance in a certain area while manipulation with a stereotype lift can enhance it [14,15]. Because mental rotation is a perceived male-stereotyped ability [16], stereotype threat effects could apply here. If the male-stereotyped cube figures often used in mental-rotation tests are partly responsible for the worse performance of females in MRTs, using more female-stereotyped material could moderate the relation of gender and performance. 

A recent study revealed that spatial abilities and perceived spatial abilities also mediate the relation between gender and math anxiety [17]. Moreover, gender differences in mental-rotation performance, perceived mental-rotation abilities (SIQ), spatial anxiety (SAM), and math anxiety (sMARS) were found. Mental-rotation performance correlated with sMARS, math performance (MATH), SAM, and SIQ, and SIQ correlated with SAM and MATH. Correlations of spatial anxiety, spatial skills, and math anxiety were found in other studies as well [18,19]. If math anxiety is affected by mental-rotation performance because of spatial anxiety, the stimulus material used in the MRT could then affect math anxiety and maybe performance in a math test as well. Stereotype threat and lift effects [14,15] caused by the rotational objects could partly be responsible for these relations. In the present study, the influence of a manipulation of the stimulus material used in the MRT on the performance in a subsequently administered math test will be investigated more closely.

Participants’ confidence in their mental rotation ability could be affected by the rotational objects and could in part be responsible for the gender differences in mental rotation and math test performance [17]. The confidence in one’s own mental rotation ability is often higher in males than in females [20]. In the present study, we studied mental-rotation performance and participants’ confidence in their own abilities in two different tests with gender-stereotyped stimuli (cube figures vs. pellet figures) as well as the effects of these tests on the performance in a subsequently administered math test. 

In the present study, we studied mental-rotation performance and participants’ confidence in their abilities in two different tests with gender-stereotyped stimuli (cube figures vs. pellet figures) as well as the effects of these tests on a subsequently administered math test. First, we expected that males outperform females in mental-rotation performance [2], and second, that these gender differences should be smaller (or absent) for pellet figures than for cube figures [12]. Third, males should estimate their MR performance higher than females [20], and fourth, males’ confidence should be more accurately related to their actual performance than females’ [21]. Fifth, assuming a stereotype lift effect by the stimulus material on subsequent math test performance, we expected participants to score higher in confidence after solving the test with stimulus material that is gender stereotyped to their own gender. Consequently, sixth, the math test performance should be better in participants after they have solved the MRT with their own gender-stereotyped stimulus material [17]. Additionally, seventh, correlations between MRT und math test performances as well as confidence will be reported. 

## 2. Method

### 2.1. Participants

One hundred and seven undergraduate students (85 female) between 17 and 40 years (*M* = 20.70, *SD* = 2.93) participated in the study. A *t*-test revealed no differences in age (*p* > 0.1) for males (*M* = 21.45, *SD* = 3.39) and females (*M* = 20.51, *SD* = 2.78). In total, 12 males and 33 females solved the P-MRT while 10 males and 52 females solved the C-MRT. 

### 2.2. Material

Two practice items and 12 test items had to be solved in the MRT [12]. Each item had a target object on the left that had to be compared to four objects on the right side. Two of the four objects were identical to the target object and had to be crossed out. The other two objects were mirrored versions of the target. All four objects were rotated in depth with angles of 45°, 90°, 135°, or 180° to the right or to the left. The C-MRT and the P-MRT were structurally equal (Figure 1). To construct the P-MRT, each cube of the C-MRT was replaced by a pellet. Reliability scores were acceptable (C-MRT: Cronbachs-alpha: 0.715, P-MRT: Cronbach’s alpha: 0.756).

As the math test, the first test (Q1) of the quantitative part of the KFT (cognitive ability test, [22]) was used, which consisted of 3 practice items and 20 test items. Each item had an amount on the left side and an amount on the right side. This was a calculation, a formula, or a geometrical dimension. Both amounts had to be compared and the larger amount had to be crossed out. With a cross in the middle, participants could indicate that both amounts were equal. 

A questionnaire collected data about participants’ confidence in their ability in the MRT with three questions (How certain have you been in your decisions in the MRT? How do you estimate your performance in the MRT? And how difficult do you think the MRT was?). All questions had to be answered on a six-point scale. To calculate a new variable for participants’ confidence, the third variable was inverted, and a mean score was calculated of all three variables. A reliability analysis revealed Cronbach’s Alpha of 0.849 for the new variable. 

### 2.3. Procedure

Participants were tested in their university classes with 30 to 45 subjects in each class. After informed consent was given, all participants started with either the C-MRT or the P-MRT depending on the class they were attending. All students of one class solved the same test, either the C-MRT or the P-MRT. First, both practice items of the MRT were solved and the right answers were discussed to ensure that all participants understood the task. Then, 12 test items had to be solved in 3 min. As a scoring method, participants got one point per item if both identical objects were crossed out and no mirrored version was apparent. Afterwards, the three questions about participants’ confidence were answered. Next, the practice items of the math test were solved and discussed before the 20 test items had to be solved in 6 min. Then, participants filled out a questionnaire about their gender and age. 

## 3. Results

### 3.1. Analysis of Guessers

Effects of the stimulus material on participants’ performance can only be analyzed if subjects understood the mental-rotation task. Therefore, guessers were eliminated from further calculations. There are six possible ways of solving one item. Therefore, a subject is identified as a guesser if he or she solved 1/6 or less items of their attempted items correctly. In total, 15 females and no males were identified as guessers. A Chi^2^-test revealed a significant deviation from an even distribution of genders and guessers (*Chi*^2^ (1) = 4.515, *p* = 0.034). Nine females were identified as guessers in the P-MRT and six in the C-MRT. For females, a Chi^2^-test for guessers and stimulus materials revealed that the distribution did not deviate from an even distribution (*Chi*^2^ (1) = 3.439, *p* = 0.064). For all further analyses, 92 participants were factored into calculations. In total, 12 males and 24 females solved the P-MRT (age: *M* = 21.14, *SD* = 3.80) while 10 males and 46 females solved the C-MRT (age: *M* = 20.39, *SD* = 2.56).

### 3.2. Analyses of Mental-Rotation Performance

**Figure 2:**
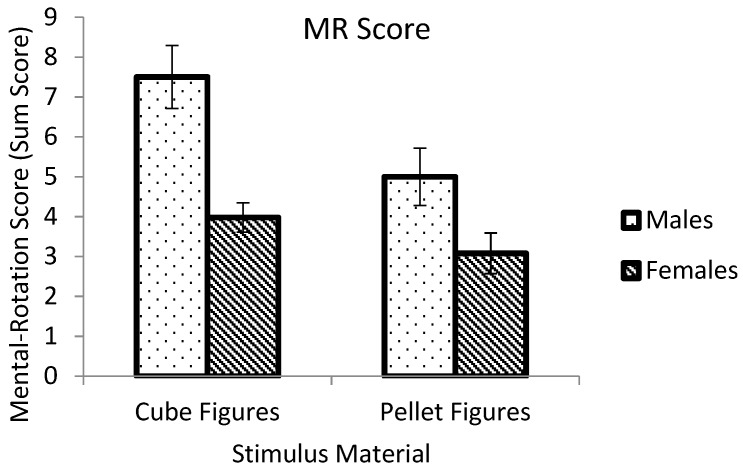
Mental-rotation score as a function of rotational objects and participants’ gender. Error bars indicate SE.

An ANOVA was calculated for the mental-rotation score, with gender and stimulus material as independent variables (Figure 2). Main effects of gender (*F* (1,88) = 19.203, *p* < 0.001, *eta*_p_^2^ = 0.179) and stimulus material (*F* (1,88) = 7.483, *p* = 0.008, *eta*_p_^2^ = 0.078) were significant. The interaction of stimulus material and participants’ gender was not significant (*F* (1,88) = 1.673, *p* = 0.199, *eta*_p_^2^ = 0.019). Differences of stimulus material were significant only for males (*F* (1,88) = 5.461, *p* = 0.022, *eta*_p_^2^ = 0.058) but not for females (*F* (1,88) = 2.024, *p* = 0.158, *eta*_p_^2^ = 0.022). Males performed better in the C-MRT than in the P-MRT. Gender differences were significant for both test versions, with a large effect for the C-MRT (*F* (1,88) = 16.321, *p* < 0.001, *eta*_p_^2^ = 0.156) and a small effect for the P-MRT (*F* (1,88) = 4.708, *p* = 0.033, *eta*_p_^2^ = 0.051).

An ANOVA was calculated for participants’ confidence in their ability in the MRT with gender and stimulus material as independent variables. Main effects of gender (*F* (1,88) = 17.211, *p* < 0.001, *eta*_p_^2^ = 0.164) and stimulus material (*F* (1,88) = 9.057, *p* = 0.003, *eta*_p_^2^ = 0.093) were significant. Males scored higher in confidence than females and participants were more confident after having solved the C-MRT than after the P-MRT. The interaction of stimulus material and participants’ gender was not significant (*F* (1,88) = 0.290, *p* = 0.592, *eta*_p_^2^ = 0.003). Males reported confidence levels of 4.40 (*SD* = 1.19) in the C-MRT and 3.44 (*SD* = 1.51) in the P-MRT while females’ scores were 3.14 (*SD* = 1.08) in the C-MRT and 2.47 (*SD* = 0.78) in the P-MRT. 

**Figure 3:**
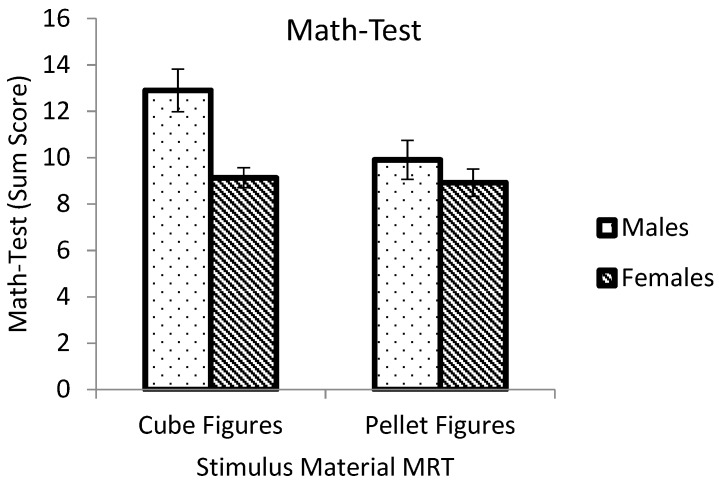
Math-test performance as a function of rotational objects of the previously solved MRT and participants’ gender. Error bars indicate SE.

An ANOVA was calculated for the math-performance score with gender and stimulus material of the previously solved MRT as an independent variables (Figure 3). Main effects of gender (*F* (1,88) = 10.951, *p* = 0.001, *eta*_p_^2^ = 0.111) and stimulus material (*F* (1,88) = 4.921, *p* = 0.029, *eta*_p_^2^ = 0.053) were significant. The interaction of participants’ gender and stimulus material failed to reach significance (*F* (1,88) = 3.693, *p* = 0.058, *eta*_p_^2^ = 0.040). Differences of stimulus material were significant only for males (*F* (1,88) = 5.766, *p* = 0.018, *eta*_p_^2^ = 0.061) but not for females (*F* (1,88) = 0.086, *p* = 0.771, *eta*_p_^2^ = 0.001). Males solved more items in the math test correctly after having solved the C-MRT than after the P-MRT. Gender differences in math-test performance were significant only in participants who had solved the C-MRT (*F* (1,88) = 13.864, *p* < 0.001, *eta*_p_^2^ = 0.136) but not after the P-MRT (*F* (1,88) = 0.950, *p* = 0.332, *eta*_p_^2^ = 0.011).

Significant correlations were found between MRT score and math-test performance, MRT score and confidence in the ability in MRT, as well as between confidence and math-test performance (Table 1). All correlations were larger for males than for females and math-test performance and confidence did not significantly correlate for females at all (*r* (70) = 0.173, *p* = 0.151). 

## 4. Discussion

Analyses of guessers and mental-rotation performance revealed higher scores for males and more female guessers. This is in line with our hypothesis and previous research [2]. A large effect of gender in the MRT with cube figures and a small effect in the MRT with pellet figures indicate that mental-rotation performance is not independent of the stimulus material [12]. However, the interaction of gender and stimulus material was not significant.

Males performed significantly better with rotating cube figures than pellet figures while there was no difference for females. Previous research found larger gender differences for more difficult material [9]. In the present study, a medium main effect of stimulus material revealed that the pellet figures seemed to be more difficult to rotate [23]. Consequently, gender differences in favor of males should have been larger in the P-MRT than in the C-MRT. As opposite results were found, this enhances the assumption that the stimulus material affected mental-rotation performance, at least for males. A stereotype lift effect [15] for males who solved the C-MRT could be a possible reason for the varying gender differences as well as a stereotype threat effect [15] for females in general. A stereotype threat effect for females could be independent of the stimulus material because mental rotation is a perceived male-stereotyped ability [16]. Hence, similar gender differences in confidence in MR ability for both test versions could not support the idea that participants rated their own performance higher when solving the test with material that was more stereotyped to their own gender. Participants reported higher scores of confidence when they solved the less difficult C-MRT than the P-MRT. Males reported higher scores than females in both test versions. Results indicate that the female-stereotyped stimulus material could not enhance females’ confidence in their own ability. It can be assumed that the uncertainty of the task itself could not be diminished by the stimulus material. Overall, a non-significant interaction showed that the confidence was not higher after solving the MRT with own-gender stereotyped objects. 

The group composition of more females than males tested in a group could have had an influence on the performance in the MRT and on participants’ confidence [24]. The group that solved the P-MRT (45 participants) was also larger than the groups that solved the C-MRT (30 and 32 participants). Distracting factors like a higher noise level could have affected the performance in the P-MRT. 

In the subsequent math test, males outperformed females only when solving the C-MRT first but not after the P-MRT, although gender differences in performance were not reported for a large norm sample (KFT, [22]). Moreover, males performed better after solving the C-MRT than after solving the P-MRT while the stimulus material of the MRT did not affect females’ math test performance. These results are only a first indication of the influence of the material in the MRT on the math test performance because the interaction of gender and material was not significant probably due to an imbalanced sample. 

Math test results indicate that males’ confidence in their own abilities could have been strengthened because of the male-stereotyped cube figures used in the C-MRT. This idea was supported by large correlations between confidence in MR ability and math test performance in males but not in females. Males’ confidence was higher than females’ and more accurately related to their actual mental-rotation performance than females’. This is in line with previous research [20,21] and our hypotheses. Males seemed to be able to judge their own performance better than females and this judgment might have affected their performance in the subsequent math test.

Sokolowski et al. [17] found similar gender differences in a mental-rotation test and perceived spatial abilities and correlations between MRT, perceived spatial abilities, and math anxiety. Results of the present study could add to these findings. A better performance of males compared to females in the C-MRT and higher scores in confidence could be a possible reason for an increase only in males’ confidence in the subsequent math test. A greater confidence could then be responsible for a better math test performance. Males in the P-MRT condition may not have benefitted more than females from the MRT because of the female-stereotyped material and therefore, their confidence in the subsequent math test was not enhanced. Females in both conditions reported lower scores in confidence in their MR ability. Hence, they could have been more insecure after having solved the MRT because spatial tasks are male stereotyped [16]. This could then have affected their confidence in the subsequent math test. 

Limitations of the present study are the uneven number of male and female participants that should be more balanced in future research and the small number of only 22 male participants. Furthermore, this imbalance could be responsible for the non-significant interactions. Therefore, the single effects should be interpreted with caution. More studies are needed that could also investigate math anxiety as a consequence of a manipulation of the rotation material.

In conclusion, the present research found that the stimulus material used as rotational objects could influence males’ performance in a mental-rotation test and in a subsequent math test. This is important, especially for diagnostic settings. If males and females are tested about their aptitude for university studies or employability, it is important to test their actual ability and not their performance in the given situation. Additionally, the stimulus material in an MRT or another spatial task should be considered carefully in order to assure test fairness, especially if subsequent tests could be affected as well. 

## Figures and Tables

**Table 1:** Correlations of MRT score, math-test performance, and confidence in mental rotation ability (MR-Ability) for all participants and for males and females separately.

	Math Test	Confidence in MR-Ability
MRT score	0.495 **	0.701 **
	Males: 0.648 **	Males: 0.779 **
	Females: 0.357 **	Females: 0.550 **
Math test	1	0.443 **
		Males: 0.806 **
		Females: 0.173

Annotation. ** *p* < 0.001.

## References

[1] Linn M.C., Petersen A.C. (1985). Emergence and characterization of sex differences in spatial ability: A meta-analysis. Child Dev..

[2] Voyer D., Voyer S., Bryden M.P. (1995). Magnitude of sex differences in spatial abilities: A meta-analysis and consideration of critical variables. Psychol. Bull..

[3] Vandenberg S.G., Kuse A.R. (1978). Mental rotations, a group test of three-dimensional spatial visualization. Percept. Mot. Skills.

[4] Quinn P.C., Liben L.S. (2014). A sex difference in mental rotation in infants: Convergent evidence. Infancy.

[5] Moè A. (2016). Does experience with spatial school subjects favour girls’ mental rotation performance?. Learn. Individ. Differ..

[6] Heil M., Jansen-Osmann P. (2008). Sex differences in mental rotation with polygons of different complexity: Do men utilize holistic processes whereas women prefer piecemeal ones?. Q. J. Exp. Psychol..

[7] Bethell-Fox C.E., Shepard R.N. (1988). Mental rotation: Effects of stimulus complexity and familiarity. Journal of Experimental Psychology. J. Exp. Psychol. Hum. Percept. Perform..

[8] Rahe M., Ruthsatz V., Jansen P., Quaiser-Pohl C. (2018). Influence of sex-stereotyped stimuli on the mental-rotation performance of elderly persons. Exp. Aging Res..

[9] Collins D.W., Kimura D. (1997). A large sex difference on a two-dimensional mental rotation task. Behav. Neurosci..

[10] Neuburger S., Heuser V., Jansen P., Quaiser-Pohl C. (2012). Influence of rotational axis and gender-stereotypical nature of rotation stimuli on the mental-rotation performance of male and female fifth graders. Proceedings of the International Conference on Spatial Cognition.

[11] Ruthsatz V., Neuburger S., Rahe M., Jansen P., Quaiser-Pohl C. (2017). The gender effect in 3D-Mental-rotation performance with familiar and gender-stereotyped objects—A study with elementary school children. J. Cogn. Psychol..

[12] Ruthsatz V., Neuburger S., Jansen P., Quaiser-Pohl C. (2014). Pellet figures, the feminine answer to cube figures? Influence of stimulus features and rotational axis on the mental-rotation performance of fourth-grade boys and girls. Proceedings of the International Conference on Spatial Cognition.

[13] Rahe M., Ruthsatz V., Quaiser-Pohl C. Influence of the Stimulus Material on Gender Differences in a Mental-Rotation Test.

[14] Steele C.M., Aronson J. (1995). Stereotype threat and the intellectual test performance of African Americans. J. Pers. Soc. Psychol..

[15] Walton G.M., Cohen G.L. (2003). Stereotype lift. J. Exp. Soc. Psychol..

[16] Hirnstein M., Andrews L.C., Hausmann M. (2014). Gender-stereotyping and cognitive sex differences in mixed-and same-sex groups. Arch. Sex. Behav..

[17] Sokolowski H.M., Hawes Z., Lyons I.M. (2019). What explains sex differences in math anxiety? A closer look at the role of spatial processing. Cognition.

[18] Ferguson A.M., Maloney E.A., Fugelsang J., Risko E.F. (2005). On the relation between math and spatial ability: The case of math anxiety. Learn. Individ. Differ..

[19] Maloney E.A., Waechter S., Risko E.F., Fugelsang J.A. (2012). Reducing the sex difference in math anxiety: The role of spatial processing ability. Learn. Individ. Differ..

[20] Estes Z., Felker S. (2012). Confidence mediates the sex difference in mental rotation performance. Arch. Sex. Behav..

[21] Cooke-Simpson A., Voyer D. (2007). Confidence and gender differences on the Mental Rotations Test. Learn. Individ. Differ..

[22] Heller K.A., Perleth C. (2000). Kognitiver Fähigkeitstest für 4. bis 12. Klassen, Revision: KFT 4-12+ R.

[23] Rahe M., Quaiser-Pohl C. (2019). Mental-Rotation Performance in Middle and High-School Age: Influence of Stimulus Material, Gender Stereotype Beliefs, and Perceived Ability of Gendered Activities. J. Cogn. Psychol..

[24] Moè A. (2018). Effects of group gender composition on Mental Rotation Test performance in women. Arch. Sex. Behav..

